# Quantitative analysis of echogenicity for patients with thyroid nodules

**DOI:** 10.1038/srep35632

**Published:** 2016-10-20

**Authors:** Ming-Hsun Wu, Chiung-Nien Chen, Kuen-Yuan Chen, Ming-Chih Ho, Hao-Chih Tai, Yu-Hsin Wang, Argon Chen, King-Jen Chang

**Affiliations:** 1 Department of Surgery, National Taiwan University Hospital, Taipei, Taiwan; 2 AmCad BioMed Corporation, Taipei, Taiwan; 3 Graduate Institute of Industrial Engineering, National Taiwan University, Taipei, Taiwan; 4 Department of Surgery, Cheng Ching General Hospital, Taichung City, Taiwan

## Abstract

Hypoechogenicity has been described qualitatively and is potentially subject to intra- and inter-observer variability. The aim of this study was to clarify whether quantitative echoic indexes (EIs) are useful for the detection of malignant thyroid nodules. Overall, 333 participants with 411 nodules were included in the final analysis. Quantification of echogenicity was performed using commercial software (AmCAD-UT; AmCad BioMed, Taiwan). The coordinates of three defined regions, the nodule, thyroid parenchyma, and strap muscle regions, were recorded in the database separately for subsequent analysis. And the results showed that ultrasound echogenicity (US-E), as assessed by clinicians, defined hypoechogenicity as an independent factor for malignancy. The EI, adjusted EI (EI_N-T_; EI_N-M_) and automatic EI_(N-R)/R_ values between benign and malignant nodules were all significantly different, with lower values for malignant nodules. All of the EIs showed similar percentages of sensitivity and specificity and had better accuracies than US-E. In conclusion, the proposed quantitative EI seems more promising to constitute an important advancement than the conventional qualitative US-E in allowing for a more reliable distinction between benign and malignant thyroid nodules.

Thyroid nodules are very common diseases^1^. The clinical importance of thyroid nodules lies primarily with the possibility of thyroid cancer, which occurs in approximately 5% of all thyroid nodules^2^,^3^. Among the imaging modalities, high-resolution ultrasonography (US) is the most sensitive diagnostic modality for the detection of thyroid nodules^4^. This modality has provided the possibility of distinguishing thyroid tumors and predicting their prognosis based on various ultrasound characteristics of the thyroid nodule^5^,^6^,^7^,^8^,^9^. However, there has been no clear consensus on the standardized terminology for thyroid US, and most of the characteristics are qualitative and subjective, making it difficult to be universally defined or applied clinically^10^.

Among ultrasound features, several studies have mentioned hypoechogenicity as an important finding suggestive of malignancy^11^,^12^. However, current studies have revealed that approximately 30–55% of benign nodules are also hypoechoic and most hypoechoic nodules are benign considering the high prevalence of benign lesions, thereby decreasing the usefulness of this US feature^13^,^14^,^15^. Marked hypoechogenicity can be a more specific and more reliable criterion for a malignant thyroid nodule than hypoechogenicity in a broader sense with a specificity of 92–94%^15^,^16^. A serious concern is that ultrasound echogenicity assessed by clinicians (US-E) has been described qualitatively and is potentially subject to intra-observer and inter-observer variability^7^. Thus, a quantitative echogenetic value (EI) more objective and measurable is desired for clinical use.

To overcome the shortcomings of subjective judgment concerning the sonographic characteristics used in diagnosis, we have proposed computerized quantification methods to characterize the calcifications, heterogeneity and vascularity to make the diagnosis more objective^17^,^18^,^19^. Additionally, the aim of this study was to collect and quantify more US information; thus, quantitative echoic indexes (EIs) are proposed to study echogenicity.

## Materials and Methods

### Participants

The Institutional Review Board of National Taiwan University Hospital approved the prospective study, and informed consent was obtained from all of the participants. The methods were carried out in accordance with the approved guidelines. There were 353 patients with 443 thyroid nodules recruited from August 2007 to February 2011 who underwent thyroidectomy because of thyroid carcinoma, a suspicious thyroid nodule, follicular neoplasm or symptomatic nodular goiter diagnosed by ultrasound and fine needle aspiration cytology (FNA) results. The diagnosis results were based on the histopathological examinations of surgical specimens that were reviewed by pathologists. Those nodules with sizes larger than the array (5.2 cm) were excluded in the image assessment. Multinodular goiters without a separable nodule under ultrasound were also excluded. Therefore, 333 participants with 411 nodules participated in the final analysis.

### Equipment and ultrasound procedures

All of the sonograms were acquired using a commercial ultrasound device (HDI 5000; Philips Healthcare, Bothell, WA) using a multifrequency linear probe (L12-5). The B-mode images, with the dynamic range of 170 dB, had widths equal to 5.2 cm, while the depths were at least 3.9 cm.

The procedure was performed with the participant in the supine position and the neck hyperextended. The images were captured using the maximum diameter of the nodule. Image analysis was conducted off-line using the Dicom format of images on a separate computer. Quantification of echogenicity was performed using commercial software (AmCAD-UT, AmCad BioMed., Taiwan). The analysis method using the software is described below in detail.

### Analysis of echogenicity

**Figure 1:**
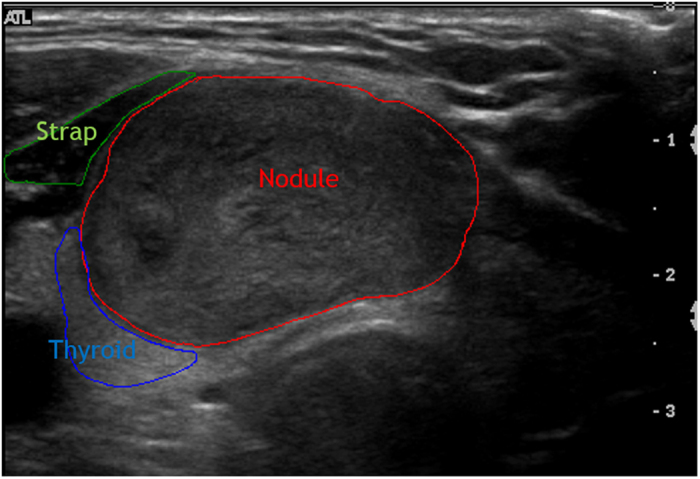
A representative image to delineate the regions of the nodule, thyroid, and strap muscle.

During the analysis of ultrasound images, the boundaries of the nodules were defined by two thyroid specialists (K. Y. Chen and M. H. Wu) without knowledge of the FNA cytology or surgical pathology results. To select the references for comparison with the nodule echogenicity^20^, the regions of the strap muscle and thyroid were also manually selected by the sonographers using computer software. The coordinates of the three defined regions, the nodule, thyroid parenchyma, and strap muscle regions, were recorded in the database separately for subsequent analysis. Examples of the images with the selected regions are shown in Fig. 1.

Next, the average gray values inside the selected regions of the nodule, thyroid and muscle, denoted as *μ*_*nodule,*_ *μ*_*thyroid*_ and *μ*_*muscle*_, respectively, were calculated. For the nodule part, the anechoic area and hyperechoic foci, clinically deemed as the cyst area and calcifications, respectively, were removed before calculation of the average. The gray values of these pixels can be regarded as outliers based on our previous study^18^, and they do not contribute to the echogenicity of the nodule. The average gray value for the remaining part of the nodule (*μ*_*nodule*_) was denoted as the echogenicity index of the nodule (EI_N_). According to the literature^15^,^16^,^21^, *μ*_*thyroid*_ and *μ*_*muscle*_ can be used as references to analyze the nodule echogenicity. The ultrasound feature of the nodule can be classified as “hypoechogenicity” when *μ*_*nodule*_ is smaller than *μ*_*thyroid*_ or as “marked hypoechogenicity” when *μ*_*nodule*_ is smaller than *μ*_*muscle*_. The differences between *μ*_*nodule*_ and *μ*_*muscle*_ and between *μ*_*nodule*_ and *μ*_*thyroid*_ were recorded, respectively, to represent the adjusted EI of the nodule and were denoted as EI_N-M_ and EI_N-T_, respectively.

**Figure 2:**
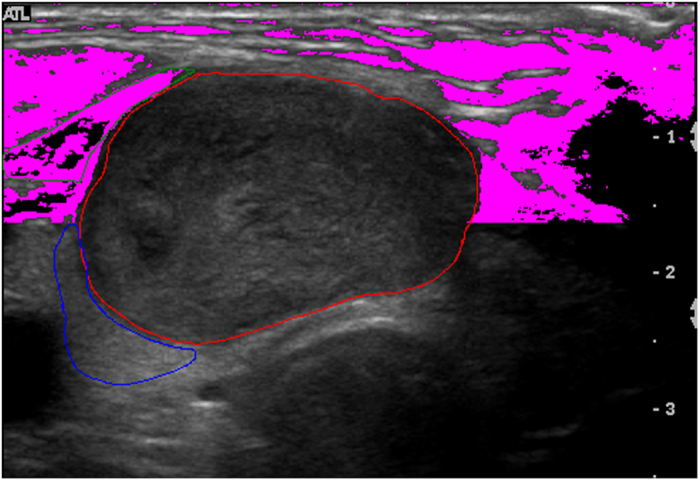
Autonomic references was calculated using the software for the same examples of images in Fig. 1.

In addition to the comparison to the manually selected references as aforementioned, an automatic calculated reference to the nodule for the echogenicity index was provided with the commercial software. Based on the anatomic knowledge that strap muscles are located mostly in the anterior region of the neck, the anterior region is defined as the area outside the contoured nodule and above the nodule center. Moreover, only those pixels in the anterior region with a gray value smaller than the average were included and defined as the outside reference to resemble the gray level similar to that of the muscle, which is generally darker than other tissue parts. Figure 2 shows the outside references calculated using the software for the same examples of images in Fig. 1.

An indicator variable *R*_*ij*_ is defined as:


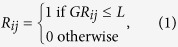


where *GR*_*ij*_ is the gray value of the pixel (*i, j*), and *L* is the average of the non-zero gray values of all pixels in the anterior region. The average gray value of the outside reference (*μ*_*ref*_) was then calculated as follows:


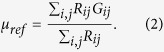


Finally, the automatic EI was obtained by


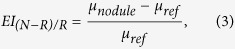


denoted as EI_(N-R)/R_, and used for further analysis.

### Statistical analysis

Statistical analysis was performed using a software package (SPSS, version 12.0 for Windows; SPSS, Chicago, III.). Fisher’s exact test was used for the comparisons of two binary variables, and Student’s t test was used for comparisons of quantitative variables. The ultrasound features were compared with the histological diagnosis results to determine the sensitivity, specificity, negative predictive value, and positive predictive value. A p value less than 0.05 was considered to indicate statistical significance. A receiver operating characteristic curve (ROC) was also generated, and the area under the curve (AUC) was calculated to determine the diagnostic performance of the quantitative EI. In addition, multiple logistic regression analysis with significant variables in the univariate logistic regression model was performed to determine independent US predictors for malignancy from the US characteristics that showed statistical significance.

Inter-observer agreement was assessed for US characteristics using the Cohen kappa statistic. The interpretation of kappa values: 0.00–0.20 indicated slight agreement; 0.21–0.40, fair agreement; 0.41–0.60, moderate agreement; 0.61–0.80, substantial agreement; and 0.80–1.00, almost perfect agreement^15^,^22^.

## Results

Of the 333 patients in our study, 269 were female, and 64 were male, with an average age of 48.37 years. The oldest patient is 81 years old and youngest is 11 years old. In total, 254 of 411 (61.8%) nodules were benign (225 were nodular goiter, and 29 were follicular adenoma), and 157 of 411 (38.2%) nodules were malignant with 7 follicular thyroid cancers (FTCs), 7 medullary thyroid cancers (MTCs), 2 anaplastic thyroid cancers (ATCs), 1 lymphoma and 140 papillary thyroid cancers (PTCs).

### Conventional ultrasound features of the benign and malignant nodules

For malignancy, 56.1% were smaller than 2 cm, 89.8% were US-E hypoechoic, 82.17% had an irregular margin, 43.31% had microcalcification, and 96.82% were heterogeneous. All of these ultrasound features showed significant differences between the malignant and benign tumors (Table 1).

### EI values of benign and malignant nodules. 

The average *μ*_*thyroid*_, *μ*_*muscle*_ and *μ*_*ref*_ values were 41.31, 18.59 and 21.72, respectively. The EI_N_, adjusted EI (EI_N-T_ & EI_N-M_) and automatic EI (EI_(N-R)/R_) values between the benign and malignant nodules were all significantly different, with lower values for malignant nodules (*p *< 0.001, AUC = 0.735, 0.7043 & 0.7698; 0.77) (Table 2).

In a univariate logistic regression analysis, either US-E hypoechoic or low EIs (cut-off set at median or zero) were statistically significant predictors of thyroid malignancy (Table 3).

US-E hypoechoic or EI_N-T_ less than zero (the same as that defined by conventional “hypoechogenicity”) was combined with other significant features for multiple logistic regression analysis to determine independent US predictors for malignancy. It showed that each of them (US-E defined hypoechoic or EI_N-T_ less than zero) was an independent predictor of thyroid malignancy (ORs: 3.51 and 3.69, respectively).

### Diagnostic performance of EIs and conventional ultrasound features

The US-E hypoechogenicity had a sensitivity of 89.8%, specificity of 31.9% and accuracy of 54% in the diagnosis of malignant nodules. EI_N-T_ less than zero had a sensitivity of 79.6%, a specificity of 52.4% and an accuracy of 62.8%. Among EIs, EI_N-M_ less than zero, as defined by conventional “marked hypoechogenicity”, had the highest accuracy at 70.3% (Table 4).

### Agreement of the Echogenicity Characteristic of the Thyroid Nodules

Among 411 nodules in our study, there are 138 nodules with the echogenicity disagreed by US-E and EI_N-T_. We evaluated the hypoechogenicity as defined by the computer system (EI_N-T_ less than zero) and clinician (US-E) and showed that they had slight agreement (kappa value 0.25). The mean |EI_N-T_| in patients with disagreement for the definition of hypoechogenicity was significantly lower than that in patients with agreement for the definition of hypoechogenicity (p < 0.0001).

**Figure 3:**
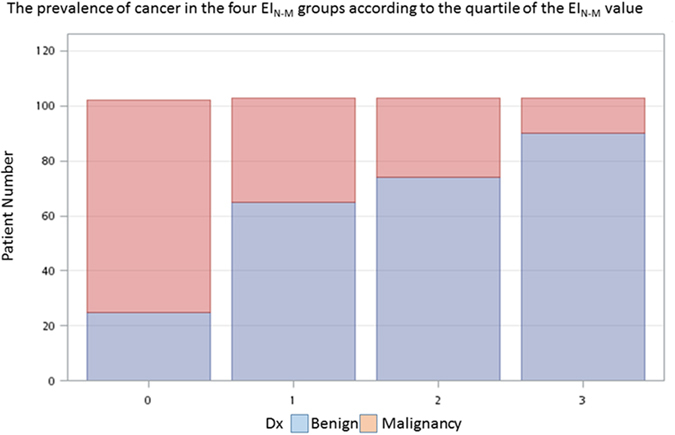
Nodules were classified into four groups according to the quartile of the EI_N-M_ value. It shows the prevalence of cancer in the four EI_N-M_ groups, and the prevalence of malignancy was significantly increased when the value of EI_N-M_ was decreased. (p < 0.001).

Because the strap muscle is thought to be a relatively consistent and reliable reference, we further classified nodules into four groups according to the quartile of the EI_N-M_ value. Figure 3 shows the prevalence of cancer in the four EI_N-M_ groups, and the prevalence of malignancy was significantly increased when the value of EI_N-M_ was decreased.

### EIs with different histology

**Figure 4:**
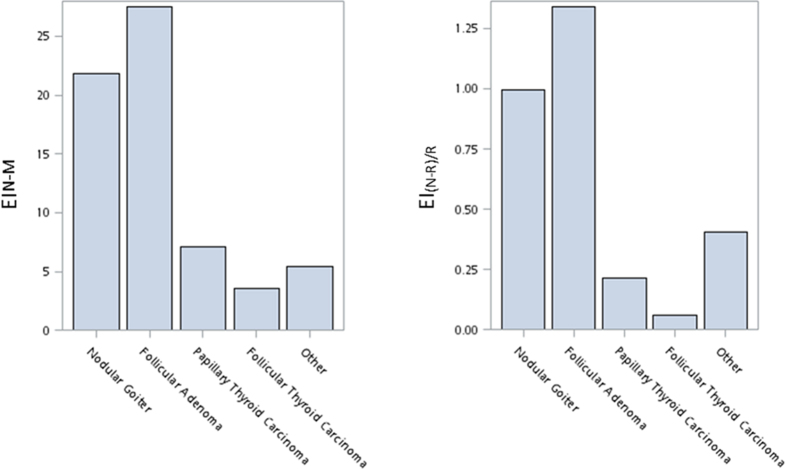
It shows that echogenicity index (EI_N-M_ and automatic EI (EI_(N-R)/R_) values for lesions classified as nodular goiter (n = 225), follicular adenoma (n = 29), papillary thyroid cancer (n = 140), follicular thyroid cancer (n = 7), and others (medullary thyroid cancers (n = 7), anaplastic thyroid cancers (n = 2) and lymphoma (n = 1).

EI_N-M_ and automatic EI (EI_(N-R)/R_) values with different histology are shown in Fig 4. The value was high in follicular adenoma and nodular goiter and low in PTC and FTC. There were significant differences between the follicular neoplasms including differences between follicular adenoma and carcinoma.

## Discussion

We proposed a computerized method to evaluate ultrasound echogenicity quantitatively. From our study, using EI values, a statistically significant difference was observed between the benign and malignant nodules. The results of this quantitative evaluation also supported the usefulness of echogenicity in the diagnosis of thyroid nodules. To our knowledge, this is the first study to report that the quantitative measurement of ultrasound echogenicity could be a helpful approach in the diagnosis of thyroid nodules using a computerized method.

The presence of microcalcifications, hypoechogenicity, irregular margins, and a solid composition with a heterogeneous pattern suggests a malignancy potential for thyroid nodules^3^,^5^,^23^,^24^. However, the sensitivity and specificity of these US findings varied in the literature^5^,^25^. Additionally, the problem regarding the use of these conventional US features is usually no standardized lexicon and terminology for characterization^7^,^13^, leading to poor reliability for the presence of some features such as the echogenicity, pattern of composition and border^7^,^26^. In addition, different qualities andlevels of clinical experience and interpretation of these findings cause variable results of the diagnostic accuracy.

We found in the current study that, among the clinician-assessed features, US-E hypoechogenicity and microcalcification, rather than irregular margin and a heterogeneous pattern, were independent predictors for malignancy. Our study found that the frequency of US-E hypoechogenicity was significantly different between benign and malignant nodules, where US-E hypoechoic nodules included the majority (89.8%) of malignant nodules. Among the US markers studied, the US-E hypoechogenicity gained the highest OR. This is consistent with findings of Moon *et al.*^15^. Additionally, EI_NT_, calculated by the computer system, when less than zero, has the same meaning as traditionally defined hypoechogenicity. Furthermore, we found EI_N-T_ to be an independent predictive factor for thyroid malignancy. We double confirmed the importance of echogenicity using qualitative and quantitative methods.

Echogenicity was traditionally assessed or described by clinician judgment. Because both benign and malignant thyroid nodules exhibited a hypoechoic pattern to different degrees, it is difficult to detect subtle differences by qualitative assessment. Most US-E hypoechoic nodules are benign considering the high prevalence of benign lesions^14^, and the comparison of echogenicity without quantification does not provide much useful information^7^,^27^.

Our EI_N-M_, when less than zero, can be classified as the traditional term “marked hypoechogenicity”. We found that EI_N-M_ (specificity: 93%; accuracy: 70.3%; ROC: 0.7698) was a more specific and reliable criterion for the diagnosis of malignant thyroid nodules than EI_N-T_ (specificity: 52%; accuracy: 62.8%; ROC: 0.7043). This result is also consistent with those in other studies that found hypoechogenicity to be highly specific for diagnosing malignant nodules^15^,^16^.

Furthermore, in the present study, because a quantitative EI_N-M_ value can be divided among different categories, we found it to be inversely correlated with the frequency of thyroid malignancy. When combined with other quantitative parameters, EI_N-M_ should improve the US characterization of nodules and help to better establish risk groups and a reporting data system for thyroid lesions in the stratification of the malignant risk of nodules^28^,^29^.

Using quantitative analysis, we found that EI_N-T_ (less than zero) had better specificity and accuracy but was less sensitive than US-E hypoechogenicity, indicating that more tumors were assessed as hypoechoic by clinicians than by the computerized system. The analysis also revealed that US-E hypoechogenicity and EI_N-T_ (less than zero) showed a slight agreement. This relatively low interviewer reliability between the clinician and computerized system was consistent with the findings of previous studies^7^,^15^. A smaller difference in echogenicity between *μ*_*nodule*_ and *μ*_*thyroid*_(low |EI_N-T_|) had a significantly higher disagreement for the definition of hypoechogenicity by the clinician and computerized system. The latter finding indicates that small subtle differences can only be differentiated by computer systems. EI seems more operator independent and more reproducible than the subjective term of US-E.

A lower EI value implies that the nodule is hypoechoic or markedly hypoechoic on the grayscale sonography, which has been defined as a suspicious sonographic feature in several guidelines^30^,^31^. It reflects the fact that a larger proportion of hypoechoic and markedly hypoechoic nodules are found in the malignant group than in the benign group. It is shown that the presence of hypoechogenicity, represented by EI_N-T_ and US-E in this study, showed a relatively high sensitivity (79.6~89.8%) but a lower specificity (31.9~52.4%) while the presence of marked hypoechogenicity, represented by EI_N-M_ in this study, was very specific (93.3%) but not sensitive (33.1%). EI_N-T_ and US-E, with which comparisons are made against the thyroid parenchyma, have a higher sensitivity than EI_N-M_, with which comparisons are made against the strap muscle, because the echo level of the thyroid parenchyma is usually much higher than that of the strap muscle. The results also agree with the previous study^15^. As for the sensitivity difference between EI_N-T_ and US-E, it is due to the disparity of the clinician perception from the computer calculation, It can be seen that the US-E is more sensitive while the EI_N-T_ is more specific to detect the malignancy. In other words, the echo level of the nodule perceived by clinicians is easier, as compared to the objectively computerized index, to be lower than the echo level of the surrounding thyroid parenchyma. In clinical situation, the interpretation of sonograms is subjective, with the inter-observer variability being unavoidable in the sonographic assessment of thyroid nodules, and sonographic interpretation is particularly affected by how much experience an operator has^1^. Operators from a single institution with different experience in thyroid imaging diagnosis have been shown to result in a significant inter-observer variability when differentiating benign and malignant thyroid nodules with grayscale sonography^32^,^33^.

With the automatic selection of the outside reference by the computer system, we can also calculate the automatic EI _(N-R)/R_, with an accuracy near 70% and an AUC near 77%, consistent with the result of EI_N-M_. Additionally, these findings suggest that manual procedures to operate the software such as selecting the ROI of reference will be more automated in the future.

Previous studies have identified certain ultrasonic features that predict follicular cancer^34^,^35^. The present study indicates that there are significant differences in the EI values between follicular adenoma and carcinoma. The result hints a possible clinical application of EI to differentiate follicular neoplasms by FNA cytopathologic diagnoses. A further prospective study will be needed to confirm the finding.

This analysis of echogenicity can be easily and quickly performed within one minute. User-friendly quantification of ultrasound image echogenicity, as described in this paper, is feasible in routine clinical practice and can be used not only for diagnoses but also as a follow-up tool for a tumor.

Although the results obtained using this method for the quantitative measurement of ultrasonic echogenicity are promising, the diagnostic performance by this single feature is still not sufficiently accurate for diagnoses. It might be improved by combining it with other ultrasonic features of computerized methods. Therefore, future studies to combine the computerized EI values with other computerized ultrasonic features are needed.

In conclusion, most conventional US markers of malignancy have been proven to be significant; however, none has ensured both high sensitivity and specificity. The proposed quantitative EI seems more promising to constitute an important advancement compared with conventional qualitative US-E in allowing for a more reliable distinction between benign and malignant thyroid nodules.

## Additional Information

**How to cite this article**: Wu, M.-H. *et al.* Quantitative analysis of echogenicity for patients with thyroid nodules. *Sci. Rep.* **6**, 35632; doi: 10.1038/srep35632 (2016).

## Figures and Tables

**Table 1:** Analysis of different US Characteristics of Benign and Malignant Thyroid Nodules.

Parameter	Benign Nodules (n = 254)	Malignant Nodules (n = 157)	*P* Value
Size			<0001
Major Diameter > = 2 cm	178 (70.08%)	69 (43.95%)	
Major Diameter < 2 cm	76 (29.92%)	88 (56.05%)	
Side			0.9007
Isthmus	5 (1.97%)	4 (2.55%)	
Left	119 (46.85%)	75 (47.77%)	
Right	130 (51.18%)	78 (49.68%)	
US-E			<0001
Hypoechogenicity	173 (68.11%)	141 (89.81%)	
Hyperechogenicity/Isoechogenicity	81 (31.89%)	16 (10.19%)	
Margin			0.0017
Well defined	81 (31.89%)	28 (17.83%)	
Irregular	173 (68.11%)	129 (82.17%)	
Microcalcification			<0001
Present	46 (18.11%)	68 (43.31%)	
Absent	208 (81.89%)	89 (56.69%)	
Echotexture			0.016
Homogeneous	24 (9.45%)	5 (3.18%)	
Heterogeneous	230 (90.55%)	152 (96.82%)	

**Table 2:** Analysis of EIs of Benign and Malignant Thyroid Nodules.

Parameter	Benign Nodules (n = 254)	Malignant Nodules (n = 157)	*P* Value
EIN	39.84 ± 16.23	27.39 ± 14.76	<0001
Adjusted EIs			
EIN-T	−1.64 ± 18.44	−13.65 ± 14.88	<0001
EIN-M	22.45 ± 15.41	6.86 ± 15.69	<0001
Automatic EIs			
EI(N-R)/R	1.04 ± 0.9	0.22 ± 0.71	<0001

**Table 3:** Results of Analysis of US Characteristics and EIs for Detection Malignant Thyroid Nodules.

Characteristic	beta Coefficient	Odds ratio	95% confidence interval	*P* value
	**Univariate**			
Major Diameter <2 cm	1.094	2.99	1.97–4.52	<0.0001
US-E Hypoechogenicity	1.4173	4.13	2.31–7.37	<0.0001
Irregular Margin	0.7688	2.16	1.33–3.51	0.002
Microcalcification	1.2396	3.45	2.21–5.41	<0.0001
Heterogenous Echotexture	1.1544	3.17	1.19–8.49	0.0216
EIN(less than median)	1.7592	5.81	3.72–9.07	<0.0001
EIN-T(less than median)	1.2091	3.35	2.2–5.1	<0.0001
EIN-M(less than median)	1.6401	5.16	3.32–8	<0.0001
EI(N-R)/R(less than median)	1.6906	5.42	3.49–8.44	<0.0001
EIN-T(less than zero)	1.4571	4.29	2.71–6.8	<0.0001
EIN-M(less than zero)	1.9319	6.9	3.81–12.5	<0.0001
EI(N-R)/R(less than median)	1.6906	5.42	3.49–8.44	<0.0001
	**Mutivariate**			
Major Diameter <2 cm	1.0615	2.89	1.84–4.54	<0.0001
US-E Hypoechogenicity	1.255	3.51	1.89–6.53	<0.0001
Irregular Margin	0.3829	1.47	0.86–2.52	0.1641
Microcalcification	1.2353	3.44	2.11–5.6	<0.0001
Heterogenous Echotexture	0.6845	1.98	0.67–5.88	0.2168
Major Diameter <2 cm	0.8452	2.33	1.47–3.7	0.0004
EIN-T(less than zero)	1.3062	3.69	2.24–6.09	<0.0001
Irregular Margin	0.5469	1.73	1–2.99	0.0511
Microcalcification	1.1994	3.32	2.03–5.44	<0.0001
Heterogenous Echotexture	0.8065	2.24	0.77–6.56	0.1414

**Table 4:** Diagnostic performance of different US Characteristics and EIs.

Characteristic	Sensitivity	Specificity	PPV	NPV	Accuracy
US-E Hypoechogenicity	0.8981	0.3189	0.449	0.8351	0.5401
Microcalcification	0.4331	0.8189	0.5965	0.7003	0.6715
Irregular Margin	0.8217	0.3189	0.4272	0.7431	0.5109
Heterogenous Echotexture	0.9682	0.0945	0.3979	0.8276	0.4282
EIN(less than median)	0.7516	0.6575	0.5756	0.8107	0.6934
EIN-T(less than zero)	0.7962	0.5236	0.5081	0.8061	0.6277
EIN-M(less than zero)	0.3312	0.9331	0.7536	0.693	0.7032
EI(N-R)/R(less than median)	0.7452	0.6496	0.568	0.8049	0.6861
